# “Home Away From Home”: A Critical Metaphor Analysis of Displaced Ukrainian Women’s Experiences With Their Canadian Hosts

**DOI:** 10.1177/23333936251353210

**Published:** 2025-07-26

**Authors:** Areej Al-Hamad, Yasin M. Yasin, Kateryna Metersky, Sepali Guruge

**Affiliations:** 1 Toronto Metropolitan University, ON, Canada; 2 University of New Brunswick, Fredericton, NB, Canada

**Keywords:** critical metaphor analysis, homestay hosting, displaced Ukrainian women, Canada

## Abstract

Displaced by the Russian invasion, Ukrainian women face complex challenges in adapting to new environments. This study explores their experiences living in homestay arrangements in Canada, drawing on Conceptual Metaphor Theory and Critical Metaphor Analysis to examine how they express displacement, adaptation, and integration. Eighteen women participated through photo-elicitation interviews, metaphor-building activities using AI-generated images, and focus group discussions. The participants created metaphors to represent their hosting experiences, which were analyzed using Charteris-Black’s framework for Critical Metaphor Analysis alongside thematic narrative analysis. Metaphors such as “The Cardboard House,” “Ferris Wheel of Photos,” and “Warm Safe House” captured their resilience, vulnerability, and evolving sense of belonging. These metaphors informed three central themes: the search for stability, safety, and peace; the process of adaptation and expressions of gratitude; and the emotional dislocation tied to homeland connections. The findings highlight the emotional complexity of homestay experiences and the need for culturally sensitive, structured hosting models. For nursing, this underscores the value of trauma- and culturally-informed care to support displaced women’s psychosocial well-being.

## Introduction

The Russian invasion of Ukraine in February 2022 led to a significant increase in homestay accommodations across Europe, where locals offered their homes to refugees (Bassoli & Luccioni, 2023). As of 2020, Canada has welcomed over 44,000 refugees and plans to admit an additional 70,000 to 80,000 refugees between 2024 and 2026 (Government of Canada, 2024). Despite Canada’s welcoming stance, many newcomers struggle with high housing costs, financial barriers, limited system knowledge, and social isolation (Schick et al., 2016). Many displaced Ukrainians in Canada face housing challenges, as some landlords demand months of rent in advance (Hasan, 2022; Weichel, 2022). The housing challenges in many host countries including Europe have led to an unprecedented rise in homestay accommodations for refugees and displaced individuals, portraying a form of hospitality where locals offer and continue to offer their homes to those displaced by conflict (Bassoli & Luccioni, 2023). While this surge is notable, the practice is not entirely new in host countries; various refugee movements over recent decades have fostered homestay practices (Bassoli & Luccioni, 2023; Hebbani et al., 2016; Logan & Murdie, 2016).

The concept of homestay accommodation particularly gained prominence and visibility during the 2015 “refugee crisis” in Europe, highlighting its role as a crucial form of support and solidarity for refugees across the continent (Bazurli, 2019). Homestay accommodations, where local residents offer their homes for limited time, involve hosts taking on the responsibility of providing a safe living space and facilitating refugees’ cultural learning and language improvement, fostering successful integration (Ran & Join-Lambert, 2020). In response to the global refugee crisis, Canada introduced public and private support systems to aid in refugee- and asylum-seeker integration. For example, Romero House facilitates settlement through volunteer-led initiatives, including short-term host matching (up to 3 months), community events, literature nights, and family-oriented gatherings (Romero house, 2024).

While both charitable activities and homestay initiatives offer physical and social support to refugees, homestays are unique in providing an intimate household setting where newcomers engage in everyday domestic life within their new society (Sirriyeh, 2013). For hosts, these initiatives offer opportunities to develop new perspectives and empathy (Alrawadieh et al., 2023). From the perspective of refugees and asylum seekers, homestay initiatives significantly improve language acquisition and intercultural adaptation by enabling daily interaction and participation in the routines of local hosts (Hebbani et al., 2016). Despite hosts’ positive intentions, the expectation of mutual support can create pressure for refugees to reciprocate, especially when hosts view them as part of the “family.” Emotions such as compassion and frustration with systemic injustices often motivate individuals to become homestay providers (Monforte et al., 2021). The imbalanced dynamics between hosts and guests put refugees at a disadvantage, especially if they cannot meet the hosts’ expectations, and they can receive labels such as “undesirable guests,” fostering negative perceptions that hinder their integration (Ran & Join-Lambert, 2020). The unconscious or conscious expectations of both sides can create further negative perceptions and strained feelings, impacting the social dynamics between hosts and refugees, where hosts’ acts of care could be perceived as an invasion of privacy by refugees (Monforte et al., 2021; Ran & Join-Lambert, 2020). Ran and Join-Lambert (2020) also highlight how privacy concerns and power imbalances in host–guest relationships can hinder refugees from expressing their needs, leading to discomfort and tension. Addressing these dynamics is essential for fostering respectful and supportive homestay experiences.

The concept of a “home” in migration is deeply shaped by gender, with women often carrying unequal burdens of caregiving, emotional labor and pressures that are intensified in contexts of forced displacement (King et al., 2022). In homestay arrangements, these roles can be amplified, as women are expected to express gratitude and conform to household norms while coping with trauma and uncertainty. Such dynamics risk reinforcing traditional gender roles and exposing women to structural vulnerabilities tied to dependency and power imbalances (Fathi & Ní Laoire, 2024; Pansary, 2025). These patterns are also evident in return migration, where women continue to face restrictive stereotypes and caregiving expectations (King et al., 2022).

The notion of hospitality is deeply embedded in social and political hierarchies, often shaped by gendered and conditional dynamics (Crossley, 2023). Rather than being universally inclusive, acts of welcoming are frequently filtered through intersecting lenses of race, nationalism, and gender (Crossley, 2023). In this context, the private home can symbolically mirror the nation-state, serving as a space where belonging is granted or denied based on sociopolitical constructs (Derrida, 2003; Ran & Join-Lambert, 2020). As such, the role of the refugee guest is not merely a domestic arrangement but one that reflects broader narratives of inclusion, power, and identity within the host society.

There are various models for hosting refugees and asylum seekers, involving either individual hosts or host families who may accommodate single individuals or entire family units. When an individual refugee is hosted by a local person, the host and the refugee can build family-like relationships because of the host’s ability to focus on one individual (Monforte et al., 2021). However, the success of this relationship can vary depending on the individual characteristics of the refugee; some may not reciprocate the affection and support provided by the host, potentially leading to negative experiences (Monforte et al., 2021). When a local family hosts a refugee family unit, unique bonds can form. Families with young children often exchange customs and traditions through homestay experiences, allowing hosted children to learn new cultural practices. These settings also expose children to values such as gender equality, as they observe shared household responsibilities within host families (Ran & Join-Lambert, 2020). Additionally, the children of the host family can gain a deeper understanding of and respect for members of other cultures by reconciling previously misunderstood knowledge (Ran & Join-Lambert, 2020).

Homestay arrangements are generally categorized as short-term (weeks to a few months) or long-term (several months to years), each with distinct advantages and challenges (Ran & Join-Lambert, 2020). Short-term stays reduce stress for both parties by offering defined timelines and manageable expectations but may limit the development of deeper relationships. In contrast, long-term stays provide stability, foster belonging, and support access to services, although they can also heighten emotional and logistical strain over time (Ran & Join-Lambert, 2020). This stability facilitates a sense of belonging and access to essential social and public services, which play crucial roles in developing independence and integrating into the new society (Ran & Join-Lambert, 2020). Tailoring duration to refugees’ needs and setting clear expectations are essential to minimize uncertainty and tension (Caron, 2019).

This study centers the experiences of displaced women using visual and narrative methodologies that align with nursing’s commitment to holistic, person-centered care (Sassen, 2023). Nurses working in diverse care settings such as community health, mental health, primary care, and transitional housing are often at the forefront of addressing the emotional, social, and health challenges that refugees encounter during resettlement (Kassam & Marcellus, 2022). Developing a deeper understanding of refugee women’s experiences in homestay arrangements can help inform the delivery of culturally responsive, trauma-informed care. By examining these experiences, this research aims to provide nurses with valuable insights for improving relational care, fostering empathy, and advocating for equitable support structures. Positioned at the intersection of participatory research, social justice, and health equity, this work is closely aligned with nursing’s broader mission to uphold human dignity and promote inclusive, community-engaged practice globally (Parry & Sivertsen, 2022).

The experiences of displaced Ukrainian women—shaped by the absence of their partners and the broader context of war—offer insights that differ significantly from those of other refugee groups. As homestay accommodation is an increasingly common residency option, it is vital to understand how refugees experience life with hosts, particularly within social, cultural, and political contexts marked by inequality and oppression. This study examines how displaced Ukrainian women perceive and construct the notion of “homestay” while living in Canadian hosts’ households.

### Theoretical Framework

This study is grounded in an interpretivist paradigm that views knowledge as socially constructed, contextually situated, and co-produced between researchers and participants (Alharahsheh & Pius, 2020). To explore the lived experiences of displaced Ukrainian women within homestay hosting arrangements, we draw on two complementary theoretical perspectives: conceptual metaphor theory (Lakoff & Johnson, 2008) and Derrida’s theory of hospitality (Derrida, 2000, 2003). Conceptual metaphor theory (CMT), developed by Lakoff and Johnson (2008), posits that metaphors are not merely rhetorical or stylistic devices but are fundamental to human thought. Metaphors shape how individuals understand, experience, and act in the world by mapping familiar, embodied experiences onto abstract concepts (Susilowati et al., 2023). CMT is grounded in an embodied ontology, which assumes that cognition is rooted in bodily and sensory experiences, and a constructivist epistemology, which holds that meaning is constructed through language, culture, and social interaction (Carpenter, 2008; Lakoff & Johnson, 2008). Critical Metaphor Analysis (CMA) complements our theoretical approach by interrogating how metaphorical language reflects and reinforces power relations, making it particularly suitable for analyzing refugee narratives within host–guest dynamics.

Anchored in interpretivist paradigms, CMA aligns with both Conceptual Metaphor Theory and Derrida’s theory of hospitality by uncovering how participants’ metaphors are shaped by, and respond to, broader sociopolitical structures and asymmetries. The use of CMT in this study aligns with previous qualitative research in migration (Arcimaviciene & Baglama, 2018) and mental health (Coll-Florit & Climent, 2019), where metaphors have been employed to access deeper meanings in the narratives of displaced individuals and refugees to illuminate how metaphors can shape migration and refugee crises (Arcimaviciene & Baglama, 2018), health-seeking behaviors and identity negotiation (Coll-Florit & Climent, 2019). In this study, CMT enables us to explore how participants use metaphors to articulate complex emotional, cultural, and social experiences of displacement, adaptation, and home-making, which often convey meanings that are difficult to express through literal language.

To examine the relational and power-laden dynamics of refugee women’s experiences in private hosting arrangements, we also draw on Derrida’s theory of hospitality (Derrida, 2000, 2003). Derrida frames hospitality as inherently paradoxical: it gestures toward openness and welcome but is always shaped by boundaries, rules, and power asymmetries (Derrida, 2000, 2003). The host maintains control over the space, and the guest is received under conditions that are implicitly or explicitly linked to behavior, gratitude, duration, and belonging (Derrida, 2000, 2003). Derrida introduces tension between unconditional hospitality, which is boundless but abstract, and conditional hospitality, which is the lived reality of structured, regulated welcome (Derrida, 2000, 2003). This theoretical lens highlights how hospitality can simultaneously offer inclusion while reinforcing exclusion or dependency.

Ontologically, Derrida’s theory assumes that relationships, space, and power are socially constructed and thus contingent on context. Epistemologically, it aligns with an interpretivist stance that prioritizes lived experience, meaning-making, and the sociopolitical dimensions of everyday interactions (Alharahsheh & Pius, 2020). Derrida’s theory of hospitality (2000) highlights the inherent contradiction between offering absolute hospitality and the need to impose limits to maintain authority over one’s own space (Derrida, 2003). Derrida also highlights the political tension between conditional and unconditional hospitality, emphasizing that hospitality typically involves certain conditions that the guest must fulfill (Derrida, 2003). This form of hospitality does not extend to those who are entirely unknown or anonymous due to the uncertainties surrounding their potential behavior (Derrida, 2000, 2003). This notion of hospitality as a mastery of space underscores the inherent asymmetry, ambivalence and power differential in the host–guest relationship (Derrida, 2000, 2003). It provides a basis for discussing the potential conditions of homestay, such as duration, legal status, host expectations, and views on how refugee women’s disparities are embodied and nested in larger social-cultural-political systems.

In this study, we applied Derrida’s framework to interpret how refugee women navigate feelings of welcome, obligation, and constraint in host–guest dynamics. By integrating CMT with Derrida’s theory of hospitality, we were able to analyze both the internal metaphoric expressions of refugee women and the external structural conditions that shape their resettlement. CMT illuminates how women cognitively and emotionally process their experiences through metaphor, whereas Derrida’s theory helps situate those expressions within a broader socio-political context of power, temporality, and conditional care. Together, these frameworks support a nuanced, multi-layered understanding of how displaced women articulate and negotiate home, care, and identity under conditions of transition and uncertainty.

## Method

We used critical metaphor analysis (CMA) to explore displaced Ukrainian women’s experiences with homestays in Canadian hosts’ households. The metaphor plays a crucial role as a figure of speech, particularly in political discourse and migration, where it is extensively utilized (Agbo et al., 2018). In this study, we employed Charteris-Black’s (2004) framework of critical metaphor analysis, which is rooted in critical discourse analysis. This framework focuses on uncovering the implicit intentions of language users, the underlying ideological structures, and the concealed power dynamics present in socio-political and cultural contexts (Agbo et al., 2018; Charteris-Black, 2004). It emphasizes the ideological and conceptual dimensions of metaphor, effectively conveying profound truths to the audience by engaging their emotions (Agbo et al., 2018). The central aim of this study is to identify, analyze, and interpret the ideological and conceptual metaphors in displaced Ukrainian women’s narratives within homestay hosting arrangements. These metaphors contribute to shaping a distinctive linguistic style, articulating speakers’ experiences, and conveying their ideologies for the purposes of rhetoric and argumentation (Agbo et al., 2018).

Photovoice was also employed as a participatory method (Wang, 2022), allowing participants to capture photographs that symbolized their lived experiences of homestay and displacement; these images were then used as visual prompts during interviews and focus groups to deepen narrative reflection and meaning-making. Analyzing the metaphors used by women can assist in uncovering deeper layers of meaning about their experiences, feelings, and challenges (Imani, 2022; Nguyen & McCallum, 2015; Susilowati et al., 2023) and how they conceptualize their temporary homes in Canada, negotiate their cultural identities, and express their sense of stability, safety and belonging. This approach can also shed light on their perceptions of host–guest dynamics, revealing insights into power relations, cultural exchanges, and emotional bonds formed in this unique context (Nguyen & McCallum, 2015; Zibin, 2022). Thus, CMA provides not only a cognitive understanding but also a window into the complex socio-political and cultural experiences of these women.

The involvement of research participants in creating metaphors as a method for gathering qualitative data through experiential activity highlights the key role the study participants can play in data generation (Vacchelli, 2018b), which can be a creative outlet for women to express their experiences and perspectives and give them a sense of agency and control over their own narratives (Drüeke et al., 2021; Stavropoulou, 2019). Metaphor and collage-making allow women to choose and use images to (re)present their experiences, which can be a cathartic and healing strategy for women who may have limited literacy or language skills and/or migration-related trauma, as it allows them to embody and negotiate their experiences of displacement in a way that is accessible to others (Vacchelli, 2018a).

### Data Collection

Following the receipt of ethics approval from Toronto Metropolitan University Research Ethics Board (REB# 2023-209), displaced Ukrainian women were recruited from September 2023 to January 2024 through network sampling (Heckathorn & Cameron, 2017), including the research team’s connections with newcomer service providers. Flyers, announcements, and social media were also used. Our study specifically focused on interviewing Ukrainian women, as they represent the majority of displaced individuals arriving in Canada. This demographic trend is largely because many of their male partners remained in Ukraine to participate in the ongoing conflict. As a result, Ukrainian women and their children have been the primary beneficiaries of homestay arrangements in Canada, making their experiences particularly relevant for understanding the dynamics and challenges of refugee hosting in this context. The participants were eligible for recruitment if they (a) were women who had entered Canada as Ukrainian refugees, (b) were aged 18 years or older, (c) had lived in Canada for at least 6 months, (d) had previous experience living with a host (but were not currently living with one), and (e) were able to recall and articulate their hosting experiences in either conversational English or Ukrainian at a secondary school level to provide informed consent and understand the questions asked during the interview. Semi- structured individual interviews that lasted 45–60 min were conducted in English via Zoom.

All recruitment and consent materials were translated into Ukrainian and reviewed with participants prior to data collection. Consent was obtained in the participants’ preferred language. Focus groups and interviews were conducted primarily in English and were audio recorded with participant consent. One team member (KM), a Ukrainian-speaking researcher fluent in both English and Ukrainian, was present to support real-time clarification and informal translation as needed. While formal childcare was not provided, the participants were welcome to bring their children. One participant brought her 2-year-old son and incorporated him into the creative process by painting his hand and embedding his handprint into her metaphor, symbolizing hope and familial continuity amid displacement. Data collection occurred over a 4-month period to allow for scheduling flexibility and participant comfort. The data collection process fostered a deeper understanding of individual and shared experiences within the homestay arrangements, and participants received a $50 gift card as an honorarium. Pseudonyms are used in the results section to protect participant confidentiality. (***See* *Table 1* *for*** the participants’ demographics.)

**Table 1.** Participant Demographics (*N* = 18).

Characteristic	Category	Number of participants
Age group	18–30 years	13
	31–40 years	2
	41–50 years	3
Marital status	Single	8
	Married/Common-law	7
	Divorced/Separated	3
Education level	Undergraduate degree	8
	Graduate degree	10
Employment status	Employed	15
	Unemployed	3
Monthly income	<$3,000 CAD	14
	>$3,000 CAD	4
Number of dependents	0–2 dependents	16
	3–5 dependents	2
Sponsorship type	*CUAET	10
	Family/Private Sponsorship	8
Duration in Canada	More than 1 year	18
Duration with host	Less than 6 months	10
	6–12 months	8

*CUAET = Canada-Ukraine Authorization for Emergency Travel.

Data collection was conducted using a multi-method qualitative approach involving photo-elicitation interviews, metaphor-building sessions, and focus groups. Eighteen displaced Ukrainian women participated in individual semi-structured interviews. Prior to these interviews, participants were asked to take or select 3 to 5 photographs that symbolized their experiences living in Canadian homestays. Clear written and verbal instructions were provided, encouraging participants to capture or select images reflecting their emotions, challenges, meaningful moments, or aspects of daily life. Ethical guidelines were emphasized, including the importance of avoiding identifiable faces and respecting privacy. These participant-generated photographs served as visual prompts during the interviews, guiding open-ended discussions on topics such as homestay dynamics, emotional and social adjustment, health and well-being, cultural adaptation, perceived power dynamics, and recommendations for future support systems.

Two weeks after the interviews, a subset of seven participants joined a half-day, in-person metaphor-building session. Each participant was invited to independently create visual metaphors that symbolized their resettlement experience. They were guided by verbal and written instructions, but no preliminary findings were shared to ensure their creative expressions remained grounded in personal reflection. While the task was individually completed, the shared setting encouraged informal interaction and peer reflection. To support this process, the research team provided a range of creative materials including stationery, colored paper, photo frames, cardboard, yarn, glue, and drawing tools. Additionally, 75 AI-generated images created via DALL·E—based on preliminary codes from the earlier interviews—were curated as optional visual prompts. These AI images were made available to participants to support and inspire their metaphor construction, particularly for those who found it challenging to represent complex or abstract ideas visually.

Following the metaphor-building session, participants engaged in a 2-hr, in-person focus group. During this session, participants shared and discussed their metaphors, collaboratively assigning names and meanings to their creations. This group dialog fostered shared interpretation and co-construction of meaning. A final virtual exit focus group was held 1 week later to reflect on and validate preliminary findings. This member-checking session ensured alignment between the research interpretations and participants’ lived experiences, allowing for corrections, elaborations, or deeper insights.

To accommodate diverse communicative styles and enhance trustworthiness, multiple expressive options were made available, including drawing, painting, collage, and written narratives. Clear guidance and illustrative examples were shared to build participant confidence and creativity. Open discussions during the focus groups further allowed participants to refine their metaphors and clarify their intended meanings. These methodological adaptations strengthened the credibility and dependability of the findings while promoting inclusivity and authentic expression.

### Data Analysis

This study adopted Charteris-Black’s (2004) CMA approach and the steps proposed by Imani (2022) for metaphor analysis. This approach has three layers, namely, metaphor identification, metaphor explanation and metaphor interpretation, providing a systematic method for analyzing the data. Data analysis unfolded in three succinct phases. Initially, metaphor identification involved the detection and selection of metaphors by the participants to depict their hosting experiences, laying the groundwork for a deeper analysis. The metaphor explanation subsequently required the participants to clarify these metaphors, linking them directly to their experiences, thereby illuminating the complex realities of their situations. The process culminated in metaphor interpretation, where the metaphors were analyzed in relation to the study’s broader objectives, revealing insights into the participants’ perceptions and emotions toward their experiences and the cultural nuances of refugee homestay experiences in Canadian host households. Metaphor titles and symbolic meanings were co-constructed during the focus group discussions immediately following the metaphor-building session, where participants explained their creative choices (e.g., colors, textures, shapes) and collectively agreed on the language that best captured the intended emotional and cultural significance of each metaphor.

All data, including transcribed interviews, focus group discussions, and digital images of participant-created metaphors, were imported into NVivo software to support systematic coding and thematic analysis. A thematic narrative analysis was conducted (Bengtsson & Andersen, 2020). Participant narratives and visual metaphors were coded inductively to identify patterns, with themes iteratively refined through constant comparison across data sources. The collected data were systematically coded by the research team, who independently identified key patterns, recurring ideas, and significant elements from the participants’ narratives (Bengtsson & Andersen, 2020). The coding process was both inductive and iterative, allowing the researchers to continually refine and adjust the codes as new data were reviewed (Bengtsson & Andersen, 2020). Once the initial codes were established, they were grouped into broader categories that reflected commonalities and connections among the participants’ experiences (Bengtsson & Andersen, 2020). Throughout the project, the research team met four times to debrief, compare coding decisions, and engage in reflexive discussions to ensure analytic consistency and strengthen interpretive validity. Through discussions and consensus among the research team, these categories were further distilled into the three overarching themes that captured participants’ experiences to address the study’s objectives. These themes represent the most salient aspects of the data, highlighting the critical elements that influence displaced Ukrainian women’s experiences in homestay arrangements.

Member checking was conducted during the virtual exit focus group, where preliminary themes and interpretations were presented to participants for feedback. Five participants affirmed the relevance of the findings and offered nuanced insights that were integrated into the final analysis. Reflexivity was employed throughout the research process via team debriefing meetings, reflexive journaling, and analytic memoing, allowing the research team to continuously examine their assumptions, positionalities, and the influence of their social locations on data interpretation. The research team’s positionalities, including their cultural backgrounds, personal experiences, migration histories, and professional commitments to equity in refugee health, inevitably influence our interactions with participants and both data collection and interpretation (Olukiun et al., 2021). This can introduce potential biases, particularly in how participants’ experiences are understood, analyzed and interpreted. To mitigate these biases, researchers have consciously reflected on their positionalities, actively sought to maintain reflexivity throughout the study (Corlett & Mavin, 2018) and employed strategies such as member checking to ensure that interpretations align with participants’ intended meanings (Corlett & Mavin, 2018).

## Results

The results are presented in two parts: first, the participant-created metaphors generated through conceptual metaphor analysis (CMA), and second, the three overarching themes that emerged from thematic analysis of the combined data sources, including interviews, metaphor narratives, and focus group discussions.

### The Developed Metaphors

The data analysis revealed that Ukrainian women used a variety of metaphors to describe their hosting experiences with Canadian hosts. The majority of these metaphors carry strong connotations that reflect not only their experiences of hosting but also their sense of displacement and the process of adapting to a new home. These connotations stem from deeply entrenched host families’ traditions, which make receiving guests or hosting refugees perceived as “a welcoming and support duty” (Birger et al., 2024), on the one hand, and “the economic struggle” of Ukrainian women living in temporary housing arrangements, on the other hand.

A total of seven participants created individual visual metaphors during the in-person session. The four metaphors presented in this section were selected for their depth, diversity, and relevance to the overarching themes of the study. All interpretations and titles of the metaphors were co-constructed by participants during the follow-up focus group discussion, where each participant described the symbolic meaning of their visual metaphor and collectively contributed to naming it. Researcher insights were used only to support contextual framing, whereas participant voices remained central to interpretation. The participants in the metaphor-building session developed the following metaphors that reflect their experiences of homestay hosting, using both visual and symbolic elements to convey complex emotions and narratives:

#### The Cardboard House: Thankful and Grateful

**Figure 1.**
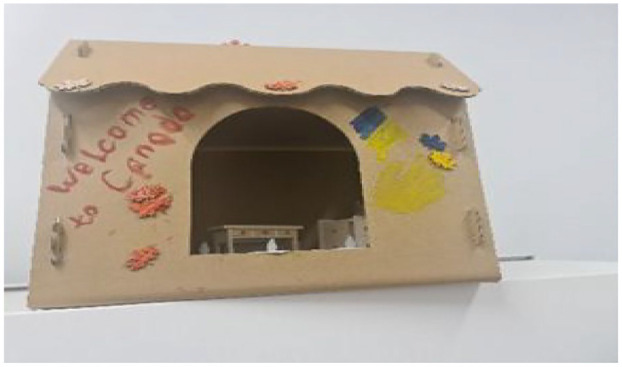
Thankful and grateful metaphor.

A small house made from cardboard, with leaves on the roof painted in the colors of the Canadian and Ukrainian flags, symbolized the participant’s appreciation for the safety found in Canada while honoring her deep-rooted connection to Ukraine, a fragile yet hopeful representation of belonging in two worlds. The house is adorned with the phrase “Welcome to Canada,” symbolizing the gratitude Ukrainian women feel toward their hosts. The baby’s hand, which gives a high five near the entrance, represents the peace and nurturing environment provided by the host. This metaphor, titled “Thankful and Grateful,” illustrates how women adapted to their new homes, creating a sense of comfort and expressing their appreciation for the support they have received. This metaphor reflects more than personal sentiments and reveals embedded ideologies and structural power asymmetries shaping the women’s resettlement experiences. Additionally, the metaphor captures both safety and fragility, aligning with neoliberal ideologies that frame refugees as self-reliant subjects responsible for their own adaptation within precarious support systems. The metaphor’s impermanence points to the temporality of care and the conditionality of welcome, reinforcing what Derrida (2000) described as hospitality marked by control and surveillance. (See Figure 1 for the developed metaphor).

#### The Wall Hanging Picture Frame Collage: Finding Hope, Peace, and Happiness

**Figure 2.**
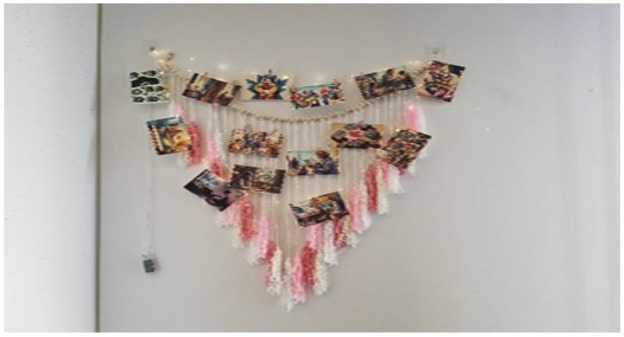
Finding hope, peace, and happiness metaphor.

Crafted from pink wool, wooden beads, and clips, this collage is integrated with lights to illuminate the photos it holds. The pink wool signifies the warmth and comfort found in Canada, whereas the wooden elements represent strength and resilience. Titled “Finding Hope, Peace, and Happiness,” this metaphor reflects the ongoing journey of Ukrainian women, capturing moments of joy and hope amidst uncertainty, as they weave together their past and present experiences. (See Figure 2 for the developed metaphor.)

#### The Ferris Wheel of Photos: Finding Support During Difficult Times

**Figure 3.**
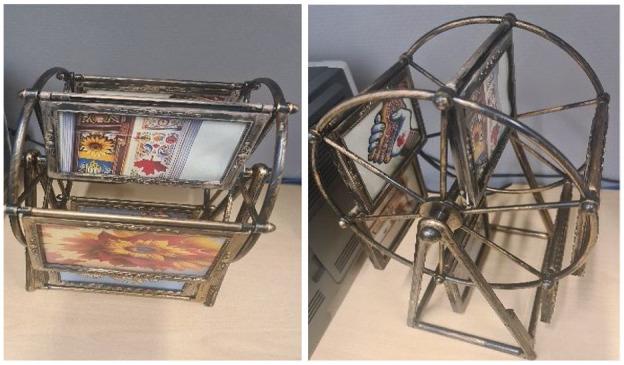
Finding support during difficult times metaphor.

A rotating display of photos that mimics the Ferris Wheel in Kyiv, symbolizing the ups and downs of the women’s experiences. The descending side of the wheel, with photos of separation and life left behind, represents the challenges and sense of loss. The ascending side, with photos of happiness and new friendships, reflects the positive aspects of their journey in Canada. Titled “Finding Support During Difficult Times,” this metaphor captures the cyclical nature of their experiences, blending memories of the past with hopes for the future. The “Ferris Wheel of Photos” metaphor evokes the cyclical and disorienting emotional labor required to remain grateful and adaptable, mirroring gendered expectations that refugee women maintain emotional resilience and composure. Through CMA, we interpret the metaphor not only as an expression of individual experience, but also as discursive construct shaped by nationalistic narratives and unspoken host expectations that regulate belonging, gratitude, and visibility. (See Figure 3 for the developed metaphor.)

#### A Wooden House and a Tree With Hanging Photos: Warm Safe House

**Figure 4.**
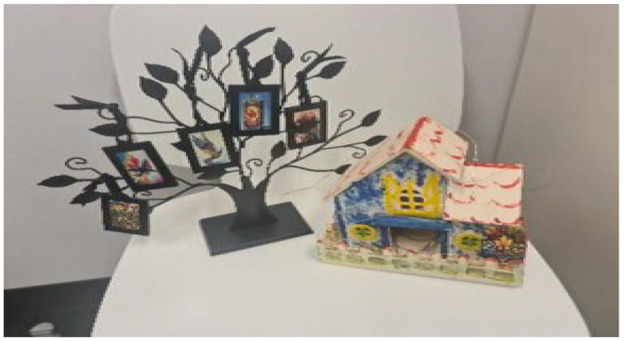
Warm safe house metaphor.

A small wooden house painted in the colors of the Ukrainian flag, symbolizing cultural identity and heritage, alongside a tree with hanging photos that represent the national tree of Ukraine, the viburnum. This metaphor, titled “Warm Safe House,” represents the fusion of Ukrainian and Canadian cultures, with the house symbolizing the merging of two distinct homelands into one safe and welcoming space. The tree, with photos that capture the women’s journey, signifies the transplantation of Ukrainian roots into Canadian soil, highlighting the complex emotions of leaving one’s homeland and integrating into a new community. (See Figure 4 for the developed metaphor.)

### Themes

The thematic analysis builds directly on the metaphors shared and interpreted by participants, as well as data from interviews and focus group discussions. The participant-created metaphors served as both visual representations of lived experience and narrative anchors that enriched our understanding of core emotional and social dynamics within homestay arrangements. The developed metaphors were co-constructed and interpreted during the focus group and helped reveal patterns of meaning that guided the identification of three overarching themes. Together, the visual and narrative data provide a holistic view of how Ukrainian women navigated displacement, adaptation, and identity within the context of private hosting. Three overarching themes were constructed during data analysis: the search for stability, safety, and peace; adaptation and gratitude; and sense of dislocation and emotional connection to the homeland.

#### Search for Stability, Safety and Peace Theme

Women expressed a deep longing for stability, safety, and peace—contrasting the disruption of war-torn Ukraine with their experiences of refuge in Canada. Viktoria’s metaphor of a “cardboard house” symbolized her feelings of being welcomed and sheltered, capturing her desire for security in a new environment. Reflecting on this sense of safety, she stated, *“For us, the warmth and security of home are captured in one word that we constantly heard, and it is simply ‘welcome to Canada’”*(Viktoria). The narrative of coming to Canada with children facing single motherhood and housing challenges underscores their search for new normalcy and safe spaces in an unfamiliar environment. The participants’ choice to include images that depict hope and peace for Ukraine’s future indicates resilient optimism, suggesting a yearning for stability and normalcy not only in their present lives in Canada but also for their homeland. In essence, Viktoria described the hanging wall photo collage as more than just a collection of photos; it is a visual narrative of their quest for stability, safety, and a sense of normalcy, hope and peace amidst the backdrop of the profound emotional turmoil brought about by displacement. Viktoria’s expression of living in two different worlds, including the safety of Canada versus the turmoil in Ukraine, highlighted participants’ longing for stability amidst the chaos.

Viktoria’s cardboard house metaphor reflected the emotional weight of leaving behind a newly renovated home in Ukraine, a space she had envisioned as her dream home but never fully inhabited due to the war. In constructing her metaphor, she recreated that vision using cardboard and miniature furniture, symbolizing both loss and the rebuilding of hope in a new context. By coloring the house with leaves in the Ukrainian and Canadian flag colors, she illustrated her transition and efforts to bridge two national identities. The phrase “Welcome to Canada,” prominently displayed in her creation, echoed her memory of arriving at the airport and represented the warmth and safety she associated with her resettlement. This emotional duality was especially evident in participants’ reflections on their early resettlement experiences and ongoing ties to Ukraine. Natalia shared, *“My son tells me, Mama, don’t watch the news today. You won’t sleep again,”* reflecting the emotional balancing act between staying connected to Ukraine and protecting her mental health. She added, *“It’s like I live in two places—my heart is still there, but my mind needs quiet here,”* capturing the ongoing inner conflict many participants expressed.

Viktoria described a powerful image from her metaphor: *“The little hands of the baby giving a high five on the cardboard house it means someone is saying, ‘You’re safe here.”* She added *“support, peace, and the feeling that someone is happy we are here.”* Other participants echoed this longing for peace and belonging through visual metaphors. *“The tree with the hanging photos it’s our life growing again,”* (Svitlana). *“The small house in the collage means shelter but also hope,”* (Liza). As Maryana put it, *“We are not just surviving here. We are trying to build something peaceful, something new.”*

Additionally, the desire for more stability and protection within homestay arrangements was echoed by several participants, who emphasized the need for formal structures to support both guests and hosts. Iryna suggested, *“If there was an agency or someone to check in, maybe things would have been clearer for both sides. We did not know what was expected, and I was always afraid to do something wrong”* Participants shared that involvement from a recognized organization or government body would provide reassurance, set clear guidelines, and protect against potential mistreatment, which could add a layer of dignity and consistency to what was often an uncertain and emotionally charged arrangement.

#### Adaptation and Gratitude Theme

There is a strong sense of adaptation to new circumstances and gratitude for the support received. Many Ukrainian women in our study mentioned feeling thankful and grateful for the help they received from Canadian hosts and the community, despite the challenges of moving and adapting to a new culture. This is evident in Olha’s statement, “*I am thankful to come to Canada and thankful on my way here . . . people have big hearts to help others.”* The narratives revealed participants’ efforts to adapt to a new culture while preserving their own cultural identity. For example, some women described blending Ukrainian and Indian cuisine, reflecting the cultural background of their host families as a way to connect and integrate. Several participants shared stories of cooking together and merging dishes, such as combining curries (Indian dishes) with traditional Ukrainian recipes. Expressions of appreciation for their hosts’ kindness and support were common and often accompanied by a strong sense of gratitude and thankfulness.

Moreover, the blend of traditions—maintaining Ukrainian customs while also adopting Canadian customs—emphasizes their journey of adaptation in their new life. The narrative of moving multiple times between different hosts while pregnant and dreaming of a reunited family in Ukraine underscores Liza’s adaptation and resilience as a refugee woman in Canada. As she shared, *“I was pregnant, moving from one house to another. . . but I always believed we would all be back together in Ukraine one day.”* This statement reflects both the hardship of displacement and her hope for familial reconnection. Similarly, Maryana described her photocollage as *“a bridge between my two worlds—my past in Ukraine and my new life in Canada,”* illustrating how visual storytelling serves as a tool for expressing identity in transition. The desire to bring back Canadian traditions to Ukraine further illustrates how their experiences in Canada are shaping their evolving sense of self and home. Sandra, for instance, spoke of her appreciation for Canadian customs: *“I love how people here send thank-you cards. I want to take that habit back with me—it is warm, it is thoughtful.”* These reflections highlight not only resilience but also the meaningful cultural exchange that can emerge through homestay experiences.

Several women described a shift from being passive recipients of care to becoming active contributors in the household. This transition often unfolds informally through daily interactions. For instance, Liza shared, *“At first, I just stayed in my room. But slowly, I started helping in the kitchen. One day, I cooked borscht, and my host said, “You are part of the family now.”* Tasks were rarely assigned; instead, women gradually offered help as they felt more comfortable. Over weeks and months, their involvement in meal preparation, cleaning, and child-minding became both a practical contribution and a source of emotional grounding. As Sandra explained, *“Helping gave me a role again. I felt less like a guest and more like a person who belonged.”*

The theme of adaptation and gratitude constructed strongly in participants’ reflections on their roles as mothers and caregivers within unfamiliar domestic settings. While women frequently expressed heartfelt appreciation for the generosity and shelter provided by their hosts, their adjustment involved negotiating divergent cultural norms around parenting, household labor, and personal autonomy—particularly in homes where Canadian women held different views on gender roles and child-rearing. Olena shared how her parenting practices evolved under the influence of her host: *“In Ukraine, I did everything for my children. Here, my host encouraged me to let them be more independent. At* first, *I felt judged, like I wasn’t a good mother. But then I started to see how my daughter grew more confident.”* This shift, while initially uncomfortable, was later reframed as a developmental opportunity for both mother and child. Similarly, Iryna noted: *“Back home, I did everything for my children—cooked, cleaned, and arranged their clothes. But here, my host told me, ‘Let them help you, they are old enough.’ It was strange at first, but now I see the value.”* However, not all changes were experienced as empowering. For some, the homestay introduced a subtle erosion of autonomy. Nataliya recalled: *“She told me how to clean the kitchen properly and where to store things. I wanted to do it my way, but I also did not want to seem ungrateful.”* Such moments reflect the delicate balance women maintained between asserting their preferences and preserving host–guest harmony. Viktoria echoed this tension: *“I didn’t want to seem ungrateful, even if I didn’t agree with the rules or felt like a guest all the time.”*

Religious practices were another area of negotiation. Svitlana recounted: *“She told my son he didn’t need to say a prayer before bed—that here, it’s not necessary. I felt like I was losing part of my culture.”* These stories illustrate the emotional and cultural complexities of adaptation, where the preservation of tradition often coexisted with enforced change. Despite these challenges, women commonly reframed their adaptations as acts of resilience. Olena stated. *“You learn to bend without breaking. It’s not easy, but I remind myself we are guests, and we were treated kindly.”* This duality—of challenge and strength—was a consistent thread in their narratives. Svitlana explained her approach as one of cultural integration rather than replacement: *“I try to build a bridge between my past in Ukraine and the life I am trying to create here.”* Visual metaphors further reflected this negotiation of identity. Maryana described her drawing of a house with two roofs—one for Ukraine and one for Canada—stating: *“That’s how my life feels now—divided but whole.”* The metaphor of the house, often colored with the flags of both nations, symbolized a dual identity forged through the experience of homestay. She added: *“It wasn’t easy to leave everything behind, but the welcome here made me feel like I belonged, even for a little while.”* Gratitude remained central to many narratives, often as a sincere acknowledgment of support. Svitlana expressed this plainly: *“We had nothing, and they opened their doors. I will always be thankful.”* Yet this gratitude was not always uncomplicated. In some cases, it coexisted with discomfort, reflecting a nuanced power dynamic that shaped participants’ sense of self and belonging.

#### Sense of Dislocation and Emotional Connection to the Homeland Theme

The majority of participants shared their sense of dislocation while simultaneously maintaining a strong connection to their homeland. Olena’s narrative of being *“physically here but our hearts are in Ukraine”* encapsulated this sentiment. The created metaphors eloquently captured the complexity of their emotional landscape. Svitlana noted that “*Even amidst smiles, there is an underlying sadness in these women’s eyes, silent evidence to the pain of being separated from their home country.”* In this statement, Svitlana referred to other Ukrainian women she had met during local community events, describing the emotional toll of displacement that, while often hidden behind smiles, remains visibly present in their expressions. Anabelle also spoke about the dual sense of belonging and longing that was poignantly described in her metaphorical representation of the Ukrainian flag and the phrase where she explicitly stated that *“the main thing we do here is seeing our country without us”*. Svitlana shared that the metaphor of the Ferris Wheel triggers memories of a similar one in Kyiv, the capital of Ukraine, and reflects a life left behind in Ukraine that embodies the dichotomy between past and present lives. Women spoke about how the metaphor of a tree representing the national tree of Ukraine in the territory of a house symbolizes the deep-rooted connection to their homeland. The depiction of a divided life—physically present in Canada but emotionally tied to Ukraine—captures their sense of dislocation. The desire to maintain Ukrainian traditions in Canada, while also embracing new Canadian customs, highlights their strong emotional connection to Ukraine and the complexity of their experiences as refugees. Several participants reflected on how living in Canadian homes as guests challenged or reshaped their traditional caregiving roles.

## Discussion

The metaphors and photo collage created by Ukrainian women to describe their homestay experiences with their Canadian hosts are not just artistic expressions; they are windows into their souls, guiding us toward a deeper understanding of Ukrainian women’s conceptualization of their notions of “refugee hosting” and “home away from home” in the Canadian context. Their metaphors serve as a vital tool in various forms of discourse, employed for purposes such as refining language, persuading, or conveying ideologies (Imani, 2022; Zibin, 2022). Several studies have focused predominantly on understanding the conceptual foundations—the source and target domains—of metaphors (Carpenter, 2008; Imani, 2022; Stickles et al., 2016; Zibin, 2022). However, metaphors extend beyond mere conceptualization; they are intrinsically linked to the intentions and ideologies underlying their usage and can be used as a form of research creation through socially engaged arts practices (Springgay et al., 2020) marked by subjectivity and analytical complexity (Imani, 2022; Nguyen & McCallum, 2015). These underlying ideologies, such as the expectation of gratitude, the moral obligation to be a “good guest,” and the gendered emotional labor embedded in caregiving and accommodation, highlight how broader cultural and social norms influence how displaced women interpret and perform their roles within homestay environments. Our study findings align with other scholars’ works where a host welcoming a guest into their home can reflect how a community regulates openness (Farahani, 2021; Luccioni, 2023; Rottmann & Nimer, 2021). Additionally, hospitality becomes a complex process that can foster both inclusion and exclusion (Luccioni, 2023).

The study findings expand the current understanding of homestays by highlighting how forcibly displaced women experience hosting not only temporary housing but also deeply relational and gendered spaces where emotional labor, gratitude, and cultural negotiation intersect (Al-Hamad et al., 2025). The study reveals that homestay arrangements are not neutral spaces of hospitality but rather sites of both support and asymmetry. This contributes to a more nuanced conceptualization of homestay in displacement contexts, including the psychosocial dynamics, power imbalances, and symbolic meanings that shape refugee women’s adaptation and well-being (Al-Hamad et al., 2025). Gender profoundly influences the challenges and dynamics within homestay arrangements, as women often bear the added pressure of fulfilling traditional caregiving roles for their children, which can intensify the strain of adapting to a new (Al-Hamad et al., 2025). Hospitality is often conditional and gendered, reinforcing hierarchical claims of migration and belonging (Crossley, 2023). The question of who is considered welcome or an (un)welcome guest becomes more entangled through processes of racialization, nationalism, and gendering, as the home itself becomes an extension of the nation (Crossley, 2023). The study findings suggest that gratitude, while genuine, may also serve as a social strategy to navigate the asymmetrical power dynamics inherent in homestay arrangements (Al-Hamad et al., 2025). It reflects both a cultural expression of thankfulness and a protective mechanism in unfamiliar environments. Recognizing this dual role of gratitude is essential in understanding the emotional labor that refugee women undertake to maintain harmony while coping with loss, transition, and adaptation.

Despite the shared challenges associated with private homestay accommodations for refugees, such as a lack of privacy, language barriers, cultural differences, and the emotional burden of adjusting to a new environment, there are notable benefits to their integration and overall well-being (Ran & Join-Lambert, 2020). By addressing these challenges, third-party involvement can provide structured support and mediation, ensuring that both hosts and refugees navigate these difficulties more effectively (Brinker, 2021). Emotional support is invaluable for refugees who have left everything familiar behind (Hebbani et al., 2016; Monforte et al., 2021; Ran & Join-Lambert, 2020). Ukrainian women find themselves not just as guests but as integral members of their new households and can foster their language skills and cultural adaptation (Monforte et al., 2021).

Gender also emerged as a critical factor shaping women’s homestay experiences, particularly through the expectation of emotional caregiving, performative gratitude, and social pressure to adapt without conflict. The women in this study frequently managed tensions within the household by self-regulating their behavior and expressing appreciation, even when discomfort or power asymmetries were present. These findings build on the literature on private hosting by highlighting how gendered norms influence not only domestic labor but also emotional expectations in host–guest relationships (Bassoli & Campomori, 2022; Bassoli & Luccioni, 2023; Boccagni & Giudici, 2022; Condon, 2023). Moreover, metaphors—such as the *Cardboard House*—expand the current understanding of shared living spaces by illustrating how safety, belonging, and instability are simultaneously felt and navigated. Rather than viewing homestay as merely a logistical solution (Bassoli & Luccioni, 2023), these metaphorical insights position it as a deeply gendered and symbolic terrain shaped by identity, care, and conditional inclusion (Bassoli & Campomori, 2022). By centering gender in our analysis, this study contributes to a more nuanced understanding of homestay as both a care practice and a site of potential harm for displaced women.

Shared living spaces blossom into a realm of identity construction (Drüeke et al., 2021), mutual respect and empathy (Gardner et al., 2022). Once they rely on the generosity of their hosts, these women now stand as pillars of support in the household. In helping with daily tasks, Ukrainian women find a sense of purpose and self-worth, an opportunity to give back, and a path to integrate into their new world (Gardner et al., 2022). These findings are congruent with those of previous studies that outline several positive impacts of homestay arrangements for refugees, such as the enhancement of language skills, the ability to address cultural knowledge gaps, improved health (Ran & Join-Lambert, 2020), the ability to exist and authority to act (Kyriakides et al., 2019), support in employment (Saksena & McMorrow, 2020), educational opportunities (Monforte et al., 2021), and strengthened social connections (Hebbani et al., 2016; Logan & Murdie, 2016). Their contributions go beyond mere chores; they are acts of gratitude, symbols of a growing bond, and steps toward building a new life. Through these interactions, refugees and hosts develop a deeper appreciation of each other’s cultures (Stavropoulou, 2019), contributing to a richer, more diverse community fabric (Logan & Murdie, 2016). This adaptation is not just a survival mechanism but rather a journey toward building a more inclusive and empathetic society.

Ukrainian women shared that by incorporating an organization or government body, there is an added layer of accountability and oversight, ensuring that hosts adhere to guidelines and treat refugees with dignity (Gardner et al., 2022). This approach not only mitigates the potential for exploitation but also provides structured support, including legal aid, counseling, and educational programs (Monforte et al., 2021). It empowers refugees with knowledge about their rights and available resources while offering hosts insights into the specific needs of their guests (Ran & Join-Lambert, 2020). Moreover, the presence of a third party offers a safety net through emergency support and conflict mediation, creating a more secure, respectful, and supportive environment for the most vulnerable members of society during their time of need.

Shared living spaces in homestay arrangements play a pivotal role in the construction and negotiation of identity for both refugees and their hosts (Merikoski & Nordberg, 2023). These environments foster daily interactions where cultural values, traditions, and personal identities are continuously exchanged and reshaped (Mahieu & Van Caudenberg, 2020). For refugees, the act of integrating into a host’s household involves navigating new social norms and expectations, which can lead to a redefinition of self-identity as they adapt to a different cultural context (Merikoski, 2022). Simultaneously, hosts may experience shifts in their own identities as they incorporate elements of the refugees’ cultures into their homes (Baggio, 2020). This dynamic interaction within shared spaces highlights the fluid nature of identity in the context of migration and underscores the importance of these spaces as more than just physical shelters but as key arenas for cultural and personal identity construction (Granata, 2020).

Although most participants were employed during the study period, many held low-wage or precarious jobs that significantly shaped their homestay experiences. Employment often introduced additional stressors, such as limited time for household responsibilities, reliance on hosts for logistical support, and ongoing financial insecurity. These dynamics sometimes reinforce feelings of dependency and vulnerability within the hosting arrangement. Furthermore, we acknowledge that participants with more negative or exploitative experiences may have been less likely to participate or to share openly due to fears of repercussion or loyalty to their hosts. As such, the findings may not fully capture the breadth of experiences among all displaced women in homestay contexts, particularly those involving conflict or coercion. Additionally, while many participants were hosted by Canadian families, we did not systematically collect data on whether hosts were of Ukrainian descent or embedded within diaspora communities. Given the cultural familiarity and shared heritage that may exist in such contexts, future research should explore how being hosted by members of the Ukrainian Canadian diaspora may uniquely influence refugees’ adaptation, emotional well-being, and sense of belonging.

### Implications

This study highlights the complex dualities Ukrainian women experience within homestay arrangements feelings of gratitude and belonging alongside moments of discomfort or dependency. While no consistent patterns of discontent or systemic failure were identified, the findings suggest opportunities for enhancing support structures, refining host–refugee matching processes, and informing future research and practice to better address the nuanced needs of displaced women in homestay contexts. While this study focuses on the specific vulnerabilities faced by Ukrainian women, we acknowledge that gendered power relations are also evident in return migration, where women face oppressive stereotypes and burdensome care duties, further highlighting the power imbalances that persist in both host and home countries (King et al., 2022). Additionally, the intersectionality of gender with other social categories, such as race, class, legal status and sexuality, must be considered when developing comprehensive and equitable migration policies (Meshram et al., 2024; Tripura et al., 2024). These intersecting factors further complicate displaced women’s experiences and must be considered in research, practice, and policy responses.

Based on the findings, we propose several considerations to enhance the homestay experience for displaced Ukrainian women. These include offering voluntary training for hosts on cultural sensitivity, communication, and awareness of refugee trauma, as well as promoting the use of clear hosting agreements to outline mutual expectations. The study illuminates the dualities women navigate such as gratitude alongside emotional vulnerability and the cyclical nature of adaptation in unfamiliar environments. While not indicative of systemic failure, these findings suggest areas where coordination and support mechanisms could be improved. In particular, third-party organizations may play a valuable role in providing resources such as legal guidance, conflict mediation, and emotional support.

Shared living spaces emerged as sites of both connection and negotiation, highlighting the need to explore how homestay arrangements can foster not hinder cultural exchange and identity reconstruction. Addressing these nuances through collaborative research, policy reflection, and responsive programing can better support both hosts and refugees. This study calls for a more comprehensive approach that not only provides physical shelter but also fosters a sense of belonging and empowerment, contributing to the creation of more inclusive and resilient communities in Canada. From a research perspective, future longitudinal research should focus on host–guest dynamics and the impact of cultural exchange on refugees’ integration. Collaboration with community organizations, non-governmental organizations, public awareness campaigns, and adequate funding for refugees’ housing support services and research are crucial for effective policy implementation. From a policy perspective, the results can inform municipal and federal settlement frameworks by calling for structured support systems, host training, and integrated health and social care models that address the psychosocial dimensions of private refugee hosting and their direct impact on women’s mental health and adaptation.

### Implications for Nursing Practice

The study findings contribute to refugee health by illuminating various dimensions of settlements, including emotional labor, cultural negotiation, and the resilience of displaced women. These dimensions are often encountered by nurses in community-based and frontline care. By understanding the layered experiences of refugee women in homestay arrangements, nurses can better advocate for trauma-informed, culturally responsive, and relational models of care that support integration, well-being, and trust-building in resettlement contexts. For nurses and health professionals, this highlights the critical need to recognize how gendered migration experiences shape health and well-being and to provide trauma-informed, culturally responsive care that addresses both physical and emotional safety within displacement contexts. The findings underscore the importance of nursing roles in supporting refugee health beyond clinical encounters, particularly in addressing psychosocial needs, fostering trust, and recognizing the impact of displacement on mental and emotional well-being. These insights are especially relevant to community health and public health nurses, who are often the first point of contact for refugee families. Integrating the lived experiences of refugee women into nursing care planning and assessment promotes person-centered, culturally safe, and equity-oriented practices, which are core principles of the nursing discipline.

## Conclusion

This study provides a nuanced understanding of how Ukrainian women perceive and construct “refugee homestay hosting” in Canada. Their metaphors vividly depict their migration, displacement, and adaptation journeys, highlighting their resilience and efforts to balance cultural identity with new opportunities. The findings emphasize the role of shared living spaces in identity construction, fostering cultural exchange and transformation for both refugees and hosts. While homestays support cultural adaptation, the study identifies gaps in host–refugee matching, emotional and psychological support, and training in cultural sensitivity, language skills, and trauma awareness. The research advocates involving third-party organizations or government oversight to strengthen homestay models, proposing clear hosting agreements and structured support. By addressing these needs, homestay programs can create more inclusive and culturally sensitive environments. Ultimately, the study contributes to academic knowledge and policy development, reflecting broader societal themes and the need for structured hosting frameworks to support displaced individuals in rebuilding their lives.
